# Antioxidant Capacity and Disease Resistance Enhanced by Dietary D-Glucuronolactone Supplementation in Chinese Soft-Shelled Turtles (*Pelodiscus sinensis*)

**DOI:** 10.3390/antiox14050534

**Published:** 2025-04-29

**Authors:** Tong Zhou, Wenyi Wu, Mingyang Xue, Yong Zhou, Hongwei Liang, Wei Liu

**Affiliations:** 1 Yangtze River Fisheries Research Institute, Chinese Academy of Fishery Sciences, Wuhan 430223, China; zhoutong@yfi.ac.cn (T.Z.); xmy@yfi.ac.cn (M.X.); zhouy@yfi.ac.cn (Y.Z.); lianghw@yfi.ac.cn (H.L.); 2 College of Animal Science and Technology, Henan University of Animal Husbandry and Economy, Zhengzhou 450046, China; wenyi1991113@163.com; 3 College of Fisheries, Huazhong Agricultural University, Wuhan 430070, China

**Keywords:** aquaculture, D-glucuronolactone, antioxidant, immune response, health, *Pelodiscus sinensis*, feeding trial

## Abstract

D-glucuronolactone (DGL), a hepatoprotective compound widely used in clinical and energy products, was evaluated for its effects on Chinese soft-shelled turtles (*Pelodiscus sinensis*) through an 8-week feeding trial with dietary supplementation (0, 200, and 400 mg kg^−1^). DGL did not alter survival or feed intake, but induced dose-dependent growth improvements, including increased final body weight, weight gain rate, specific growth rate, and muscle/liver glycogen, alongside reduced feed conversion ratio and muscle and liver fat. Serum analysis showed decreased activities of alanine aminotransferase, aspartate aminotransferase, alkaline phosphatase, and reduced low-density lipoprotein cholesterol, total cholesterol, and triacylglycerols. Antioxidant indices revealed elevated catalase and superoxide dismutase (SOD) activities in serum and intestine, coupled with reduced malondialdehyde, though hepatic SOD activity declined. Histologically, 400 mg kg^−1^ DGL alleviated liver lesions without impacting intestinal morphology. Molecular analyses demonstrated upregulated muscle *mTOR* signaling genes (*mTOR*, *IGF1*, *S6K1*) but downregulated hepatic/intestinal *mTOR* and *IGF1* expression. DGL also suppressed inflammatory cytokines (*TNF-α*, *IL-1β*, *IL-10*) in liver and intestine. Challenge tests with *Aeromonas hydrophila* confirmed the enhanced disease resistance in DGL-supplemented turtles. These findings highlight DGL’s potential as a nutritional strategy to enhance growth, antioxidant capacity, and health in intensive turtle farming.

## 1. Introduction

The Chinese soft-shelled turtle (*Pelodiscus sinensis*) stands as one of the most highly prized species in East and Southeast Asia, finding extensive use in food production, traditional medicine, and the pet trade [1]. In recent years, it has emerged as a significant economic component of China’s aquaculture industry, with an annual production exceeding 400,000 tons in 2023, while the output value has exceeded CNY 20 billion (China Fishery Statistical Yearbook 2024) [2]. However, the sustainable and healthy development of *P. sinensis* aquaculture faces significant challenges due to frequent disease outbreaks over the past few decades [3]. Studies have indicated that the suboptimal health state caused by the deteriorating aquaculture environments, imbalanced feed nutrition, and drug misuse constitutes key contributing factors to immune suppression and pathogen susceptibility in *P. sinensis* [4,5,6]. Notably, the liver—a vital organ integrating functions in nutrient metabolism, drug detoxification, and immune regulation [7]—plays a pivotal role in modulating the suboptimal health status of *P. sinensis* [8]. Its functional integrity shows significant correlations, with both compromised growth performance and heightened disease susceptibility in this species [5,9,10,11]. Hence, strategies aimed at improving hepatic health and immune resilience in *P. sinensis* are of significant importance for optimizing aquaculture sustainability.

Assessment of hepatic health status in animals constitutes a complex systematic engineering project [12] involving a multi-scale approach, from molecular to organ levels. Molecular indicators include gene expression related to metabolism, detoxification, and immunity, along with antioxidant enzyme activities and lipid peroxidation markers [13]. Cellular assessments focus on hepatocyte morphology and serum biomarkers [14]. Organ-level indicators encompass macroscopic liver characteristics, such as color [15] and size [16]. Hepatic health is also influenced by its bidirectional relationship with intestinal function, affecting overall resistance to pathogens in aquatic species [17]. This integrated approach provides a comprehensive assessment of liver health in animals. However, a comprehensive assessment of liver health is only the first step; developing targeted intervention strategies is even more critical.

D-glucuronolactone (DGL), a naturally occurring compound, is ubiquitously present in living organisms and chemically defined as D-(+)-glucofuranurono-6,3-lactone [18,19]. In animal physiology, DGL undergoes hepatic metabolism primarily via hydrolysis into glucuronic acid [18], a bioactive metabolite with multifaceted physiological functions including detoxification, antioxidant activity, and anti-inflammatory properties [19,20]. Consequently, DGL has been extensively employed in clinical settings for managing liver disorders, ranging from chronic and acute hepatitis to cirrhosis and other liver diseases, with demonstrated efficacy in mitigating drug-induced liver injury incidence [21,22,23].

The current literature on the application of DGL as a sole feed additive in animal nutrition is rather limited and predominantly focuses on poultry. In laying hens, dietary supplementation with 140 mg kg^−1^ DGL has been shown to enhance hepatic enzyme activity, ovarian follicle development, and eggshell quality during peak production periods [24]. A patented formulation further suggests that 280 mg kg^−1^ DGL reduces hepatic lipid deposition in hens, potentially through the modulation of fatty acid metabolism [25]. By contrast, research in aquatic species is scarce, with only one study demonstrating hepatoprotective effects in teleost fish: Shi et al. reported that 200 mg kg^−1^ DGL improved lipid metabolism, antioxidant capacity, and immune function in tilapia (*Oreochromis niloticus*), highlighting its potential for aquatic applications [26].

As an aquatic reptile, *P. sinensis* exhibits distinct physiological and metabolic characteristics compared to teleost fish, including seasonal hibernation behavior with hepatic lipid accumulation serving as the primary energy reserve during dormancy [27]; bimodal respiration utilizing pulmonary ventilation supplemented by cutaneous and buccopharyngeal gas exchange during prolonged submergence [28]; hepatic expression of complete ornithine–urea cycle enzymes enabling ureotelism [29], contrasting with the predominant ammoniotelic excretion in most fish species; and unique urea excretion through oral mucosa rather than conventional reptilian cloacal pathways [30]. These traits suggest that the effect of DGL on detoxification, lipid metabolism, and antioxidant defense in *P. sinensis* may differ mechanistically from those observed in fish.

Therefore, this study aimed to evaluate the effects of dietary DGL supplementation on growth, liver and gut health, antioxidant status, gene expression, and disease resistance in *P. sinensis*. The findings of this study will serve as a scientific basis for the application of DGL in aquaculture reptiles, with a particular focus on hepatoprotective strategies in intensive aquatic farming systems. Given that liver health is a critical issue in fish aquaculture [31], the insights from this study could potentially inform strategies for mitigating liver disorders in other fish species as well.

## 2. Materials and Methods

### 2.1. Ethic

This study was approved by the Animal Experimental Ethical Inspection of Laboratory Animal Center, Yangtze River Fisheries Research Institute, Chinese Academy of Fishery Sciences (NO. YFI2024liuwei03).

### 2.2. Experimental Diets

This study utilized a single-factor experimental design to assess the effects of dietary DGL supplementation. The feed formula was designed according to the national standard of the People’s Republic of China (GB/T 32140-2015 formula feed for soft-shelled turtle *P. sinensis*) [32]. The primary raw materials included fish meal, blood meal, soybean meal, expanded soybean, corn gluten meal, and α-starch. Based on previously reported efficacious levels in *O. niloticus* (200 mg kg^−1^) [26] and poultry studies (up to 280 mg kg^−1^) [25], experimental diets were formulated by supplementing with 0 (control, C), 200 (G200), and 400 (G400) mg kg^−1^ pharmaceutical-grade DGL (purity ≥ 98%, Zhucheng Haotian Pharmaceutical Co., Ltd., Zhucheng, China). The former employs a conservative dosing strategy, while the latter explores the potential for dose optimization. Experimental diets were created by supplementing with 0 (Control, C), 200 (G200), and 400 (G400) mg kg^−1^ pharmaceutical-grade DGL (purity ≥ 98%, Zhucheng Haotian Pharmaceutical Co., Ltd., Zhucheng, China). Diet formulation and proximate composition are presented in Table 1.

### 2.3. Experimental Soft-Shelled Turtle and Culture Management

The male *P. sinensis* used in the experiment was obtained from Xijia Agricultural Development Co., Ltd. in Bengbu, Anhui Province, China. After being transported back to the base, the experimental turtles were temporarily cultured in a recirculating water aquaculture system for three weeks to acclimate to the environmental conditions. During this accumulation period, they were fed the C diet. Following acclimatization, 180 healthy turtles with vigorous vitality, intact skin, and uniform sizes (average weight: 110.77 ± 1.70 g) were selected. These turtles were randomly assigned to 9 tanks with the original holding system, with 20 turtles per tank (L × W × H: 100 cm × 100 cm × 35 cm). The tanks were randomly allocated into three treatment groups, each receiving one of the three experimental diets described in Section 2.2. Each dietary treatment was replicated in three tanks. The feeding trial lasted for 8 weeks. During this period, turtles were fed twice daily (at 08:00 and 17:00) at a feeding rate of 2–3% of body weight. After 30 min, any uneaten feed was collected, and feed intake was calculated based on the feed dissolution rate. Throughout the experiment, the water temperature was maintained at 28–31 °C, ammonia nitrogen was less than 0.5 mg kg^−1^, nitrite was less than 0.1 mg kg^−1^, and the pH was between 6.5 and 7.2.

For further molecular investigations, approximately 0.1 g of liver, intestinal, and muscle tissues were collected from three additional turtles per tank, flash-frozen in liquid nitrogen at −80 °C for future research purposes. Subsequently, all remaining tissues were combined and stored at −20 °C for body composition analysis. Finally, the remaining turtles in each tank were counted, and their body weights were recorded.

### 2.4. Sample Collection

After the feeding experiment, three turtles per tank were randomly selected and euthanized for morphometric measurements and biochemical analyses. Each turtle was rapidly measured on an ice-cooled surface within 2 min to determine trunk length (longest carapace point), trunk width (widest carapace point), trunk height (highest carapace point), and body weight. Following decapitation, blood samples were collected and centrifuged at 3000 rpm (4 °C, 10 min) to separate serum, which was then stored at −80 °C for biochemical and antioxidant analyses. The liver was carefully dissected and weighed. Portions of liver and intestinal tissues at the same site were fixed in 4% paraformaldehyde for histological analysis, while parallel samples were homogenized (1:9 *w*/*v*) in ice-cold 0.9% NaCl, centrifuged (4000 rpm, 4 °C, 10 min), and the supernatant stored at −80 °C for antioxidant and digestive enzyme assays. To assess nutritional composition, the remaining liver and leg muscle tissues from each tank were pooled and stored at −20 °C. Additionally, liver and carapace/plastron regions were analyzed for L*, a*, and b* color values using a WSC-1B colorimeter (Shanghai Yidian Scientific Instrument Co., Ltd., Shanghai, China).

### 2.5. Proximate Composition Analysis

The moisture, crude protein, crude fat, and ash contents of the collected samples were determined according to the national standard of the People’s Republic of China: GB/T 5009.3-2003 (Determination of Moisture in Foods) [34], GB/T 5009.5-2003 (Determination of Protein in Foods) [35], GB/T 5009.6-2003 (Determination of Fat in Foods) [36], and GB/T 5009.4-2003 (Determination of Ash in Foods) [37]. Briefly, moisture content was measured by drying samples at 105 °C for 4 h until a constant weight was achieved. Crude protein was analyzed using a fully automated Kjeldahl nitrogen analyzer (Model K-360, BÜCHI Instrument, Flawil, Switzerland), with protein content calculated as nitrogen content 6.25. Crude fat was extracted via the Soxhlet petroleum ether extraction, and ash content was determined by incinerating samples at 550 °C for 12 h in a muffle furnace.

### 2.6. Serum Biochemical, Antioxidant, and Digestive Enzyme Assays

Serum biochemical indices were analyzed using Sysmex reagent kits (Sysmex Wuxi Co., Ltd., Wuxi, China) and a Sysmex-800 fully automated biochemistry analyzer (Sysmex Infosystems, Kobe, Japan). The measured parameters included: aspartate aminotransferase (AST, determined by the UV malate dehydrogenase method, code: 290505, 290506), alanine aminotransferase (ALT, determined by the UV lactate dehydrogenase method, code: 290503, 290504), glucose (GLU, measured using the hexokinase method, code: 290523), total cholesterol (TCHO, assayed by the CHOD-PAP method, code: 290536, 290537), triacylglycerols (TG, assayed by the GPO-PAP method, code: 80943, 80944), high-density lipoprotein cholesterol (HDLC, measured using the direct method, code: 290544), low-density lipoprotein cholesterol (LDLC, also measured using the direct method, code: 84106, 84107), total protein (TP, determined by the Biuret method, code: 290518), albumin (ALB, assayed using the bromocresol green method, code: 290515), and alkaline phosphatase (ALP, measured using the AMP buffer method, code: 290501, 290502).

Superoxide dismutase (SOD, code: A001-1-2) and catalase (CAT, code: A007-1-1) activities, malondialdehyde (MDA, code: A003-1-2) content in serum, liver, and intestine, serum lipopolysaccharide (LPS, code: H255-1-2), and lipase (code: A054-1-1), protease (code: A080-1-1), and α-amylase (code: C016-1-1) activities in hepatic and intestinal tissues were measured using commercial assay kits (Nanjing Jiancheng Bioengineering Institute, Nanjing, China) according to the manufacturer’s protocols. All enzymatic activities and metabolic concentrations in tissues were normalized to total protein content, which was determined using the Bradford method, with bovine serum albumin (BSA) as the standard [38].

### 2.7. Liver and Intestinal Histology

Liver and intestinal tissues fixed with 4% paraformaldehyde (24 h) were processed using standard histological techniques to generate semi-continuous sections (5 μm thickness), which were then stained with hematoxylin and eosin. Images were acquired using an OLYMPUS CX41 microscope coupled with an OLYMPUS DP73 digital camera (Tokyo, Japan). Four representative fields per tissue sample were captured per turtle, following methodologies described by [39,40]. Histopathological evaluation was performed using semi-quantitative criteria outlined in Table 2.

### 2.8. Real-Time PCR Analysis

Total RNA was extracted from liver, muscle, and intestine, and subsequently purified using the VeZol reagent kit (Code: R411-01, Vazyme Biotechnology Co., Ltd., Nanjing, China). To eliminate potential genomic DNA contamination, the extracted RNA was treated with RNA-free DNase. The reverse transcriptase cDNA was synthesis using a reverse transcriptase cDNA synthesis kit (Code: R223-01, Vazyme Biotechnology Co., Ltd.) following the manufacturer’s instructions.

Quantitative real-time PCR (RT-qPCR) was performed on a CFX96TM real-time PCR detection system (Bio-Rad, Hercules, CA, USA). Each 20 μL reaction contained 10 μL 2× SYBR^®^ Premix Ex Taq^TM^ II (code: Q311-02, Vazyme Biotechnology Co., Ltd.), 7.8 μL sterilized double-distilled water (ddH_2_O), 1 μL diluted cDNA (1:10), and 0.6 μL each of forward and reverse primer (10 μM). The RT-qPCR program consisted of an initial activation step at 95 °C for 30 s, followed by 40 cycles of 95 °C for 15 s, 60 °C for 30 s, and 15 s at 60 °C. The amplification efficiency was between 95% and 105%, with the standard curve correlation coefficient exceeding 0.98. β-actin was used as the internal gene for normalization. Following PCR, a melting curve analysis was conducted to confirm the specificity of the amplified products. Relative quantification of target gene expression was calculated using the 2^−ΔΔCt^ method. Primer sequences for qPCR are provided in Table 3.

### 2.9. Challenge Test

The challenge test was conducted using the *Aeromonas hydrophila* T3 strain [43,44] which was originally isolated from *P. sinensis*. The experiment followed the protocol established by Zhang et al. [44]. For the challenge test, 10 turtles per tank were randomly selected and marked dorsally with a non-invasive identifier. The selected turtles were immersion-challenged with a bacterial suspension at 1.35 × 10⁶ CFU mL^−1^. Daily mortality rates were recorded throughout the 10-day challenge period, while routine feeding protocols were maintained unchanged.

### 2.10. Calculation of Performance Parameters and Statistical Analysis

The following parameters were calculated using standard equations:

Survival rate (SR, %) = 100 × final number/initial number

Weight gain rate (WGR, %) = 100 × [final mean weight (g) − initial mean weight (g)]/initial mean weight (g)

Specific growth rate (SGR, % day^−1^) = 100 × [ln (final mean weight (g)) − ln (initial mean weight (g))]/rearing period (days)

Feed conversion ratio (FCR) = dry feed intake (g)/[final weight (g) + dead weight (g) − initial weight (g))]

Feed intake (FI, % body weight day^−1^) = 100 × total feed intake (g)/[(final weight (g) + dead weight (g) + initial weight (g))]/2 × rearing period days]

Hepatosomatic index (HSI, %) = 100 × liver weight (g)/body weight (g)

Condition factor (CF, g cm^−3^) = 100 × body weight (g)/[body length (cm)]^3^

All statistical analyses were conducted using SPSS 22.0 (IBM Corp., Armonk, NY, USA). Experimental data are presented as mean ± standard error (SEM) based on three independent replicates. One-way analysis of variance (ANOVA) was used for intergroup comparisons, followed by Tukey’s post hoc test for multiple comparisons. Statistical significance was determined at *p* < 0.05. Data visualization was created with GraphPad Prism 9.0 (GraphPad Software, San Diego, CA, USA).

## 3. Results

### 3.1. Growth Performance and Feed Utilization

As shown in Table 4, dietary DGL supplementation demonstrated no significant effects on survival rate (SR) or feed intake (FI) (*p* > 0.05). However, dose-dependent improvements in growth parameters were observed. Final body weight (FBW), weight gain rate (WGR), and specific growth rate (SGR) progressively increased with higher dietary DGL levels, accompanied by gradual reductions in feed conversion ratio (FCR). Compared to the control group (C), the G400 treatment group exhibited statistically significant differences in these indices (*p* < 0.05), while the G200 group showed non-significant trends (*p* > 0.05).

### 3.2. Morphometric Parameters

No significant differences were observed among groups for hepatosomatic index (HSI), condition factor (CF), or trunk length-to-width (TL/TW), trunk length-to-height (TL/TH), and trunk width-to-height (TW/TH) ratios (*p* > 0.05, Table 5).

### 3.3. Body and Hepatic Chromaticity

As shown in Table 6, dietary supplementation with DGL had no significant impact on the color intensity parameters (L*, a*, b*) of the carapace or plastron (*p* > 0.05). However, in hepatic tissues, while lightness (L*) and redness (a*) remained consistent across all groups, yellowness (b*) demonstrated a dose-dependent decrease. Specifically, the G400 group exhibited significantly lower yellowness values compared to the control group (*p* < 0.05).

### 3.4. Body Composition

**Figure 1:**
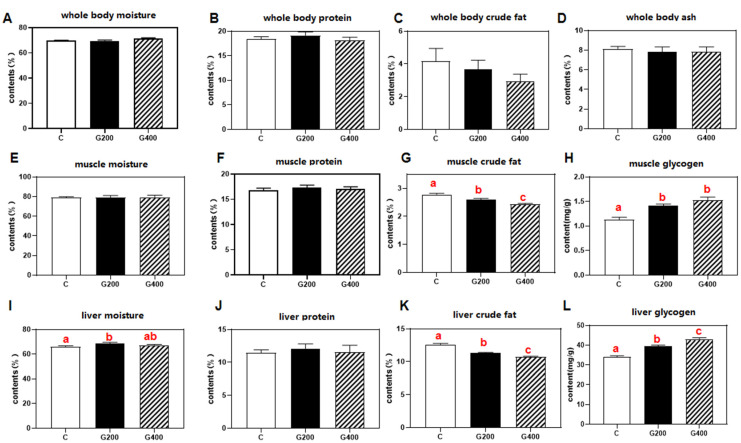
Effects of DGL on body composition of *P. sinensis* (**A**–**L**). The contents of moisture, protein, crude fat, and ash in whole body, muscle, and liver. Mean values in the same line with different superscript letters are significantly different (*p* < 0.05). Values are represented as the mean ± SEM (n = 3).

Proximate composition analysis revealed no statistically significant differences in moisture, crude protein, crude fat, or ash content in whole-body composition across experimental groups (*p* > 0.05) (Figure 1). However, dietary DGL supplementation induced tissue-specific alterations, characterized by significant reductions in crude fat content and enhanced glycogen deposition in both muscular and hepatic tissues (*p* < 0.05).

### 3.5. Hepatic and Intestinal Digestive Enzymes

**Figure 2:**
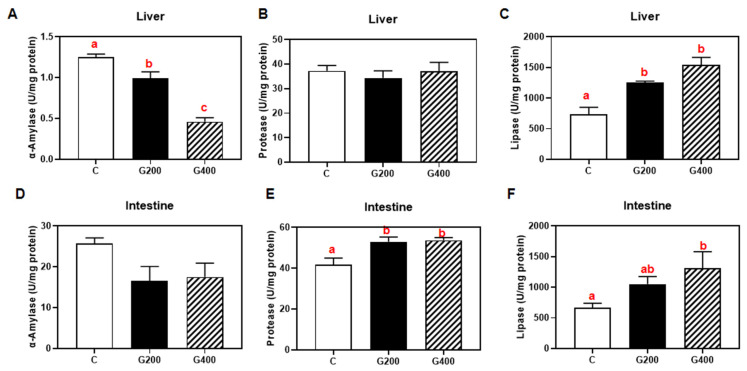
Effects of DGL on hepatic and intestinal digestive enzymes of *P. sinensis* (**A**–**F**). The contents of α-amylase, protease, and lipase in the liver and intestine. Mean values in the same row with different superscript letters are significantly different (*p* < 0.05). Values are represented as the mean ± SEM (n = 3).

As shown in (Figure 2), in the liver, α-amylase activity decreased dose-dependently (*p* < 0.05), whereas lipase activity increased markedly (*p* < 0.05), with protease activity remaining unchanged. In the intestine, protease and lipase activities significantly increased with higher DGL supplementation levels (*p* < 0.05), while amylase activity remained unaffected (*p* > 0.05).

### 3.6. Serum Biochemistry Parameters

As detailed in Table 7, dietary DGL supplementation led to dose-dependent reductions in multiple serum biomarkers. ALT, AST, and ALP activities, along with LDLC, HDLC, TC, TP, TG, and LPS levels, decreased progressively with increasing DGL levels. The G400 group exhibited significant reductions in all measured parameters (*p* < 0.05), while the G200 group showed statistically significant decreases in ALP activity and HDL-C, TC, and TG levels (*p* < 0.05). No significant differences were detected in serum glucose or albumin concentrations among groups (*p* > 0.05).

### 3.7. Antioxidants Index

**Figure 3:**
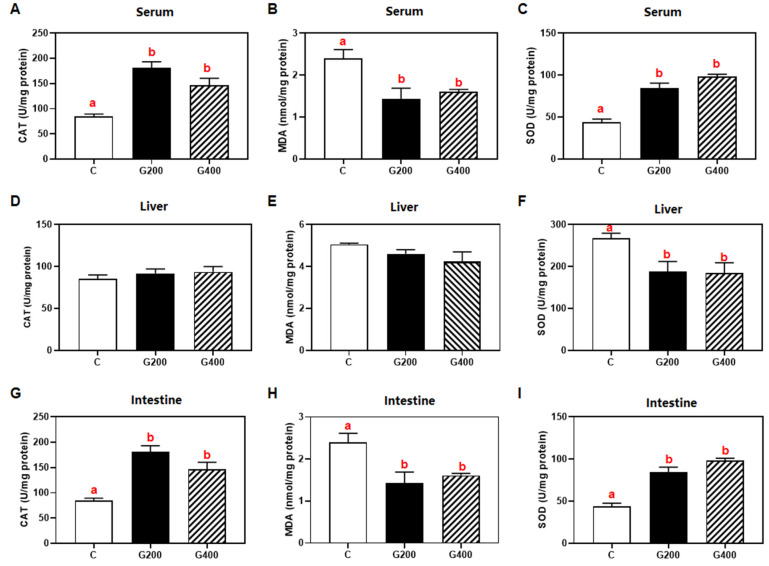
Effects of DGL on the antioxidant index of *P. sinensis* (**A**–**I**). The contents of CAT, MDA, and SOD in serum liver and intestine. Mean values in the same row with different superscript letters are significantly different (*p* < 0.05). Values are represented as the mean ± SEM (n = 3).

Dietary DGL supplementation triggered tissue-specific antioxidant responses (Figure 3). In the serum and intestine, catalase (CAT) and superoxide dismutase (SOD) activities significantly elevated as the dietary DGL level increased, whereas malondialdehyde (MDA) levels significantly decreased (*p* < 0.05). In the liver, SOD activity was significantly reduced compared to the control group (*p* < 0.05), while CAT activity and MDA levels remained statistically unchanged (*p* > 0.05).

### 3.8. Liver Histology

**Figure 4:**
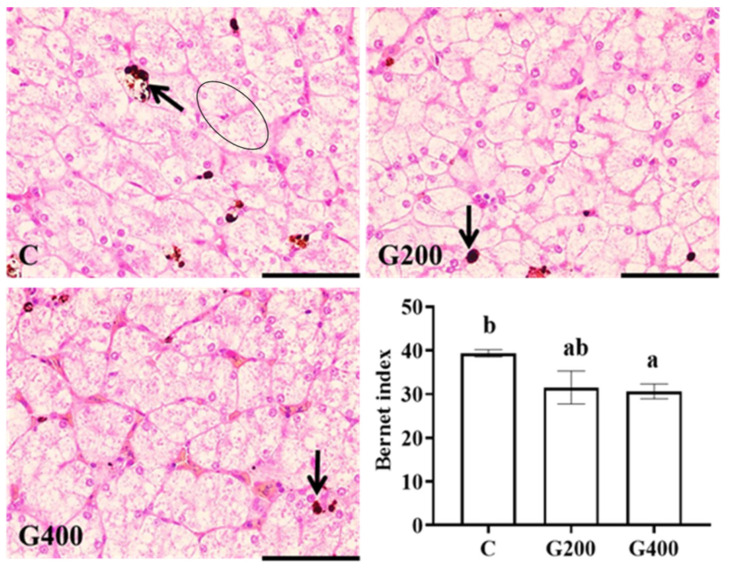
Effects of DGL on liver histology of *P. sinensis*. The arrow indicates lipofuscin deposition. The ellipse indicates the fusion of liver cells. Different superscript letters indicate significant differences between each treatment (*p* < 0.05). The line represents 50 μm. Values are represented as the mean ± SEM (n = 3).

The hepatic histological assessment results are presented in Figure 4. While inflammatory cell infiltration was occasionally observed across all experimental groups, hepatocyte vacuolation was consistently present. In particular, the control group showed lipofuscin deposition and loss of hepatocellular boundaries, which were alleviated by DGL supplementation. Semi-quantitative analysis indicated that DGL supplementation exhibited a trend toward reducing the liver lesion index, with the G400 group showing significantly lower values when compared to the C group (*p* < 0.05). Additionally, no statistically significant difference was found in the hepatic lesion index between the G200 and G400 groups (*p* > 0.05).

### 3.9. Intestine Histology

**Figure 5:**
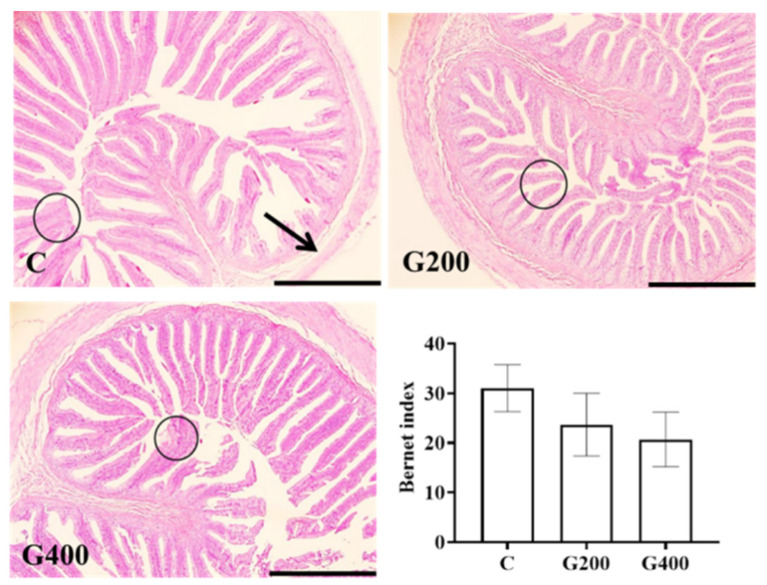
Effects of DGL on intestinal histology of *P. sinensis*. The circle indicates hyperplasia and hypertrophy of the mucosal layer, and the arrow indicates desquamation of muscle fibers. The line represents 500 μm. Values are represented as the mean ± SEM (n = 3).

Histological evaluation revealed consistent intestinal architecture across all experimental groups predominantly characterized by intestinal villus hyperplasia and concurrent hypertrophy. Additionally, occasional epithelial cell necrosis and desquamation of muscle fibers in the muscular layer were also observed (Figure 5). Semi-quantitative analysis showed a trend toward a reduction in the injury index with DGL supplementation, although the differences between groups were not statistically significant (*p* > 0.05).

### 3.10. The Relative mRNA Levels of Growth-Related Gene Expression in Three Tissues (Liver, Intestine and Muscle) of P. sinensis

**Figure 6:**
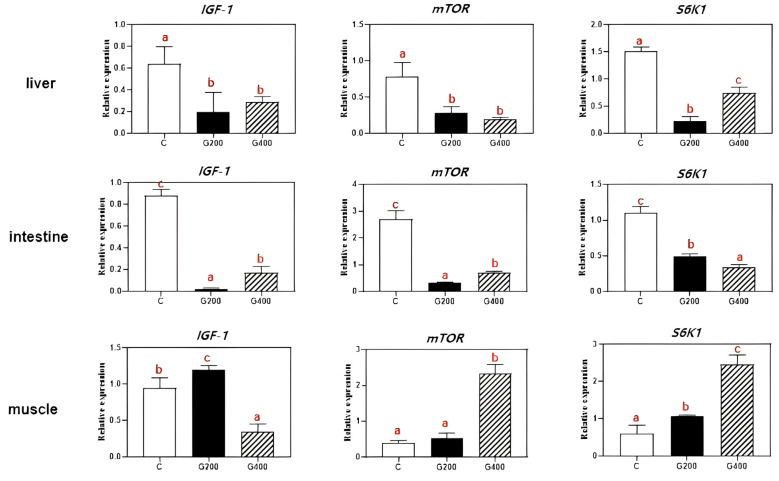
Effects of DGL on liver, intestine, and muscle growth-related genes expression of *P. sinensis*. Different superscript letters indicate significant differences between each treatment (*p* < 0.05). Values are represented as the mean ± SEM (n = 3).

The effects of dietary DGL on the mRNA levels of growth-related genes in three tissues of *P. sinensis* are shown in Figure 6. In the liver, the expression of *IGF-1* and *mTOR* expression was significantly downregulated (*p* < 0.05) in both G200 and G400 groups compared to the C group, with no significant differences between G200 and G400 (*p* > 0.05). However, the expression of *S6K1* tended to increase with DGL supplementation, reaching the highest level in the G400 group, which was significantly higher than in both the C and G200 groups (*p* < 0.05). In the intestine, the expression of *IGF-1*, *mTOR*, and *S6K1* were markedly suppressed in both DGL-treated groups (*p* < 0.05) compared to the C group. In muscle, the expression of *IGF-1* was highest in G200, significantly greater than in C and G400 (*p* < 0.05), with G400 showing the lowest expression among the three groups. However, the *mTOR* and *S6K1* expression exhibited progressive upregulation with increasing DGL supplementation, with G400 showing the highest expression levels (*p* < 0.05).

### 3.11. The Relative mRNA Levels of Inflammatory Gene Expression in Three Tissues (Liver, Intestine, and Muscle) of P. sinensis

**Figure 7:**
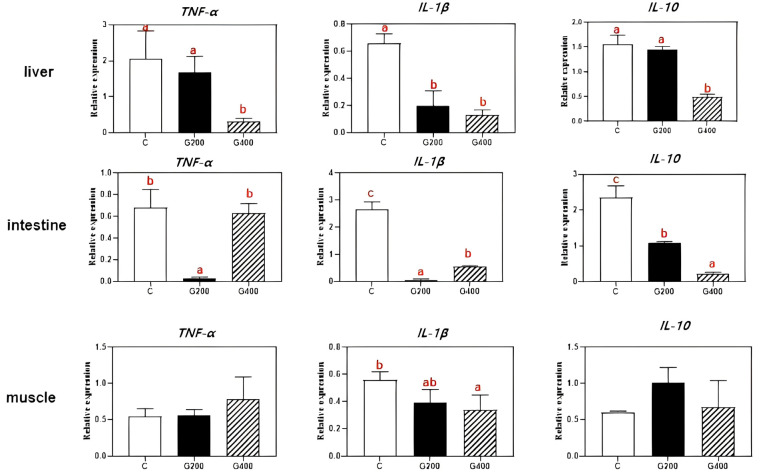
Effects of DGL on liver, intestine, and muscle inflammatory gene expression of *P. sinensis*. Different superscript letters indicate significant differences between each treatment (*p* < 0.05). Values are represented as the mean ± SEM (n = 3).

As presented in Figure 7, in the liver, the expression of *TNF-α* and *IL-10* was significantly downregulated in the G400 group compared to both the C and G200 groups (*p* < 0.05). However, no significant differences were observed in *TNF-α* and *IL-10* expression between C and G200 groups (*p* > 0.05). In the intestine, the mRNA expression of *TNF-α* and *IL-1β* declined with increasing dietary DGL supplementation and then upregulation in G400 groups, but the *TNF-α* expression showed no significant difference between C and G400 groups (*p* > 0.05). Additionally, the *IL-10* expression exhibited a dose-dependent decline, with both G200 and G400 groups showing significantly lower levels than the C (*p* < 0.05), and G400 groups displaying the lowest expression (*p* < 0.05). In muscle, *TNF-α* expression showed an increasing trend with higher DGL supplementation, but no significant differences among the groups (*p* > 0.05). In contrast, the *IL-1β* expression followed a declining trend, with the lowest expression observed in the G400 group (*p* < 0.05). The *IL-10* expression initially increased and then declined as dietary DGL content increased; however, no significant differences were observed among groups (*p* > 0.05).

### 3.12. Challenge Test

**Figure 8:**
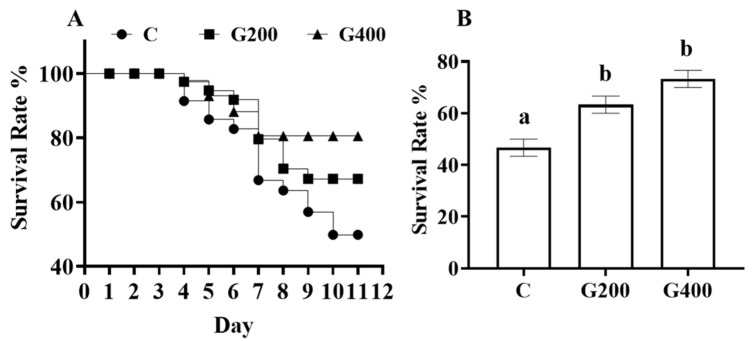
(**A**) Cumulative mortality statistics for *P. sinensis* infected with *A. hydrophila*; and (**B**) the survival rate of each group at 10 days post-challenge. Error bars represent the standard error of the mean (n = 3). Different superscript letters indicate significant differences between each treatment (*p* < 0.05).

The effects of dietary DGL supplementation on the survival rate of *P. sinensis* following *A. hydrophila* challenge is illustrated in Figure 5. The cumulative mortality curve (Figure 8A) shows that survival rates declined progressively across all groups, with the C group exhibiting the earliest and steepest mortality decline, stabilizing at approximately 45% survival by day 10. In contrast, the G200 and G400 groups displayed delayed mortality onset and improved survival, with G400 demonstrating the highest survival rate. The final survival rates at 10 days post-challenge (Figure 8B) indicated that both G200 and G400 groups exhibited significantly higher survival rates compared to the C group (*p* < 0.05), though no significant difference was observed between G200 and G400 (*p* > 0.05). These results suggest that dietary DGL supplementation enhances disease resistance in *P. sinensis*, reducing mortality following *A. hydrophila* infection, with a dose-dependent protective effect, particularly in the G400 group.

## 4. Discussion

### 4.1. Effects of DGL on Growth and Metabolism in Chinese Soft-Shelled Turtles

In the present study, *P. sinensis* was fed at approximately 2% of body weight per day (BW d^−1^) (Table 4), resulting in an exogenous DGL intake of 4 mg kg^−1^ BW d^−1^. This dosage is significantly below the reported no-observed-adverse-effect limit (NOAEL) of 1000 mg kg^−1^ BW d^−1^ [45], confirming the safety of DGL supplementation. Our findings demonstrated that dietary DGL supplementation exerted positive effects on the growth performance of *P. sinensis*, with the benefits increasing as the dosage increased. This observation is consistent with many studies on functional feed additives for aquatic animals [46,47]. To our knowledge, limited research has explored DGL effects on aquatic species; a study in tilapia revealed similar growth improvement trends at 200–400 mg kg^−1^ dietary DGL [26]. Additionally, a rodent model study documented body weight gains in thioacetamide-induced liver fibrosis rats administered 75 BW d^−1^ DGL [19]. Beyond growth improvement, our study further identified the beneficial effects of DGL on feed utilization, accompanied by enhanced intestinal protease and lipase activities (Figure 2). These findings align with previous observations in tilapia [26], suggesting that DGL may enhance nutrient digestion and absorption via enzymatic regulation.

Following its in vivo conversion to glucuronic acid, DGL modulates carbohydrate metabolism through dual mechanisms: (1) inhibition of hepatic amylase activity [48], directly suppressing glycogenolysis; and (2) potential activation of glycogen synthase [49], thereby increasing hepatic glycogen storage [50,51]. Our experimental data corroborate this mechanism, demonstrating reduced hepatic amylase activity, decreased blood glucose levels, and elevated liver glycogen content. Concurrently, DGL administration significantly improved lipid profiles, evidenced by reduced serum TG and TC (Table 7), accompanied by decreased hepatic/muscular lipid deposition and enhanced hepatic lipase activity. This lipid-modulating effect may stem from DGL’s capacity to alter fatty acid chain length and saturation in lipid molecules, thereby destabilizing lipid structures to promote triacylglycerol hydrolysis while inhibiting lipogenesis [25].

The observed upregulation of *IGF1*, *mTOR*, and *S6K1* gene expression in muscle tissue (Figure 6) aligns with established mechanisms where IGF-1 activates mTOR signaling to stimulate protein synthesis and cellular growth [52,53,54]. This signaling cascade, mediated through S6K1 activation [55], likely drives the enhanced myofiber development [56]. Contrastingly, the suppressed expression of these genes in hepatic and intestinal tissues may be attributed to glucuronic acid-mediated glycogen conservation. By inhibiting glycogenosis, glucuronic acid creates a cellular energy deficit that inactivates the nutrient-sensitive mTOR pathway [57,58]. This metabolic reprogramming hypothesis is further supported by decreased serum protein levels (Table 7), though precise molecular interactions require elucidation through targeted proteomic analyses.

### 4.2. Effects of DGL on Antioxidant Capacity and Tissue-Specific Responses in Chinese Soft-Shelled Turtles

In the present study, DGL exhibited significant antioxidant effects in serum and intestinal tissues, as evidenced by increased CAT and SOD activities alongside reduced MDA levels (Figure 1). These results indicate its capacity to alleviate oxidative stress by enhancing free radical scavenging and boosting antioxidant enzyme activity. Paradoxically, hepatic SOD activity decreased significantly without CAT/MDA alterations. This dichotomy may arise from either compensatory activation of endogenous antioxidant systems (e.g., the glutathione system) through alternative pathways, or preferential engagement of glucuronic acid, a metabolic derivative of DGL, in specific antioxidant mechanisms that suppress reactive oxygen species (ROS) generation [59]. Such metabolic reprogramming could trigger feedback inhibition of SOD activity [60], suggesting tissue-specific modulation of redox homeostasis by DGL.

### 4.3. Effects of DGL on Hepatic and Intestinal Histology in Chinese Soft-Shelled Turtles

The reduction in serum ALT, AST, and ALP activities serves as a critical indicator for evaluating attenuated hepatic injury [61]. In the present study, the activities of these enzymes decreased significantly with increasing DGL supplementation, particularly in the G400 group (Table 7), demonstrating its efficacy in improving liver health in *P. sinensis*. Additionally, hepatic yellowness (reflected by b* value), a recognized marker of pathological hepatic alterations [62], was significantly lower in the G400 group, indicating that their liver color was closer to a healthy state. Furthermore, histopathological analysis revealed that the control group exhibited typical signs of hepatic steatosis and oxidative damage, including lipofuscin deposition and loss of hepatocyte boundaries (Figure 4) [63,64]. In contrast, these pathological features were attenuated in the G200 and G400 groups, with the G400 group showing the lowest hepatic lesion index. These findings collectively corroborate the hepatoprotective mechanisms of DGL, mediated through specific biochemical pathways. While *P. sinensis* was chosen as a model species due to its ecological and economic importance, our findings may have broader implications for improving liver health across various fish species in aquaculture systems.

In the present study, intestinal villus hyperplasia and hypertrophy were observed across all experimental groups, likely representing a physiological adaptation in aquatic animals fed formulated diets. Such morphological changes may enhance digestive and absorptive capacity under conditions of high nutrient intake [65]. Notably, dietary DGL supplementation showed a trend toward reduced intestinal lesion indices, suggesting potential improvements in gut health. The serum lipopolysaccharide (LPS) levels, predominantly derived from the outer membranes of Gram-negative gut bacteria [66], serve as potent immune activators capable of triggering systemic inflammation [67]. The observed reduction in serum LPS in the G400 group (Table 6) is attributed to the detoxification function of DGL, which eliminates endogenous and exogenous toxins as well as neurotransmitters in the hepatic portal circulation [68]. This process reduces LPS translocation into the bloodstream, thereby supporting the protective effects of DGL on intestinal barrier integrity.

### 4.4. Effects of DGL on Inflammatory Response and Disease Resistance in Chinese Soft-Shelled Turtles

Inflammation, a critical component of immune surveillance and host defense [69], exerts beneficial effects at moderate levels, but becomes detrimental when excessive. TNF-α and IL-1β, two pivotal pro-inflammatory cytokines, play central roles in initiating and sustaining inflammatory responses [70]. Conversely, IL-10 exhibits context-dependent dual regulatory functions in inflammation [71]. This study revealed that dietary DGL supplementation significantly suppressed *TNF-α*, *IL-1β*, and *IL-10* expression in the liver, intestine, and muscle of *P. sinensis*. The most pronounced inhibitory effects occurred in hepatic tissues, with dose-dependent suppression of these cytokines (particularly TNF-α) being observed in the G400 group. This suggests that DGL alleviates inflammation primarily through downregulating pro-inflammatory mediators, with the liver emerging as its key anti-inflammatory target organ. Notably, an intriguing dose–response divergence manifested in intestinal tissues: while the G200 group showed the lowest *TNF-α/IL-1β* expression levels, these inflammatory markers rebounded significantly in the G400 group. This paradoxical phenomenon may reflect gut microbiota-mediated immunomodulation, lower-dose DGL may exert enhanced anti-inflammatory effects by optimizing gut microbiota structure to more effectively suppress pro-inflammatory cytokine expression [68,72].

*A. hydrophila*, a ubiquitous aquatic microorganism, represents one of the most frequently isolated pathogens from diseased aquatic species [73]. Infection by this bacterium causing hemorrhagic septicemia has been identified as a predominant threat in Chinese soft-shelled turtle aquaculture [74]. In the present study, dietary supplementation with 200–400 mg kg^−1^ DGL enhanced survival rates in pathogen-challenged turtles (Figure 8), demonstrating potent immunostimulatory effects.

## 5. Conclusions

**Figure 9:**
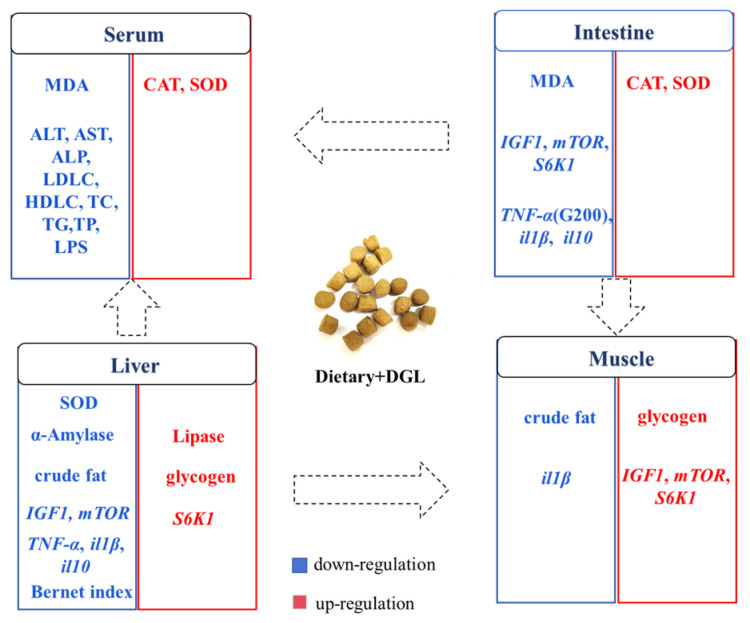
Graphical overview of the main effects of dietary DGL on antioxidant capacity and health status in *P. sinensis*. Blue and red words represent down- and upregulation of enzyme activities, genes, and contents.

Our study represents the inaugural demonstration that dietary supplementation with 200–400 mg kg^−1^ of DGL significantly enhances the growth performance and feed efficiency of *P. sinensis*. This beneficial effect can be attributed to the activation of the IGF1/mTOR/S6K1 signaling pathway in muscle tissue, which stimulates protein synthesis and myofiber growth. Additionally, DGL optimizes carbohydrate metabolism by suppressing hepatic amylase activity and boosting glycogen storage. Remarkably, DGL exhibits robust antioxidant and anti-inflammatory properties, as manifested by elevated serum and intestinal SOD/CAT activity, reduced oxidative stress (evidenced by lower MDA levels), and a dose-dependent decrease in pro-inflammatory cytokines (TNF-α, IL-1β) in tissues. The hepatoprotective effects of DGL are particularly noteworthy, including the mitigation of hepatic lipid accumulation, reduction in serum ALT/AST levels, and restoration of liver color. Importantly, DGL supplementation at 200–400 mg kg^−1^ significantly enhances the resistance of *P. sinensis* to *A. hydrophila* infection. Collectively, these findings suggest that DGL holds great promise as a functional feed additive for promoting sustainable aquaculture practices (Figure 9). Indeed, many studies on feed additives for aquatic animals have observed dose-dependent effects, where low doses promote growth and physiological performance, while high doses inhibit or have no effect [75,76,77]. In our trial, the highest dose was only designed to reach 400 mg kg^−1^, and whether a dose-dependent effect exists remains to be determined. In the future, we will continue to explore the impact of higher doses of DGL on aquatic animals.

## Figures and Tables

**Table 1:** Formulation and proximate composition of the diets.

Ingredients	Experimental Diets		
	C	G200	G400
Fish meal	505.00	505.00	505.00
Blood cell meal	30.00	30.00	30.00
Brewer’s yeast	30.00	30.00	30.00
Soybean meal	50.00	50.00	50.00
Puffed soybeans	50.00	50.00	50.00
Corn gluten meal	30.00	30.00	30.00
α-starch	200.00	199.80	199.60
Glucurolactone	0.00	0.20	0.40
Bentonite	15.00	15.00	15.00
Celite	20.00	20.00	20.00
Premix	50.00	50.00	50.00
Ca(H_2_PO_4_)_2_	15.00	15.00	15.00
50% Choline chloride	5.00	5.00	5.00
Proximate composition (%, dry matter)			
Moisture	15.26	14.36	16.28
Crude protein	43.22	43.51	43.45
Crude lipid	5.75	5.72	5.69
Ash	14.42	14.40	14.35

Premix (per kg of diet) according to Zhang et al. [33]: retinal acetate, 5000 IU; calciferol, 5000 IU; tocopherol, 700 mg; menadione, 50 mg; thiamin, 70 mg; riboflavin, 200 mg; cyanocobalamin, 50 mg; Ca pantothenate,320 mg; nicotinic acid, 400 mg; folic acid, 20 mg; inositol, 800 mg; ascorbic acid, 1 g; biotin, 20 mg; choline chloride, 2 g; MgSO_4_, 5 g; KCl, 5 g; NaCl, 2 g; CuSO_4_·5H_2_O, 50 mg; ZnSO_4_·7H_2_O, 280 mg; FeSO_4_·7H_2_O, 300 mg; MnSO_4_·5H_2_O, 1 mg; KI, 0.5 mg; NaSeO_3_·5H_2_O, 0.4 mg; CoCl_2_, 0.1 mg.

**Table 2:** Bernet index.

Tissue		Changes Observed	Factor of Importance (w)
Liver		Inflammatory cell infiltration	2
		Melanin-containing macrophages	1
		Necrosis	3
		Sinusoidal congestion	1
		Loss of cell limit	2
		Cellular vacuolation	1
Intestine	Mucosal layer	Pithelial cell necrosis	3
		Hyperplasia/hypertrophy	2
	Lamina propria	Hyperplasia/hypertrophy	2
	Muscular layer	Hyperplasia/hypertrophy	2
		Desquamation of muscle fibers	3
		Inflammatory cell infiltration	3
		Tissue disintegration and necrosis	3

Bernet index = Σ factor of importance (w) × score (α). w quantifies the reversibility potential of tissue damage using a 3-tiered scale: 1 (mild reversible damage), 2 (moderate damage, reversible upon removal of the stimulus), 3 (severe pathological importance, typically irreversible lesions leading to partial or complete loss of organ function). α evaluates lesion severity through a 4-grade classification: 0 (no change), 2 (low incidence), 4 (moderate incidence), and 6 (severe).

**Table 3:** Primers used for real-time PCR analysis.

Gene	Sequence (5′-3′)	GC %	Tm °C	Amplicon Size (bp)	GenBank Accession
*β-actin*	F: TGAGCTTCGTGTAGCACCTG	55.00	60.04	252	XM 006134860
	R: AGGATGGCATGGGGTAAAGC	55.00	60.11		
*TNF-α*	F: TCCTCCGGCACATCATCTTG	55.00	59.82	116	XM 014575959
	R: GTACCACACTTCGGTCTCGG	60.00	60.11		
*IL-1β*	F:TCCAACACCAAGTGAGGCTG	55.00	60.18	249	NM 001317048
	R: ACTCAAACTGGGTGGTGTCC	55.00	59.82		
*IL-10*	F: ACAGGAAATATGGGGAAGGACG	55.00	59.83	126	KT 203380
	R: AAGATTTAAACTGAGGTTCTGGAAG	55.00	57.25		
*IGF1*	F: CAAGCCACCCAAATCTGCAC	55.00	60.04	105	NM 001286920
	R: CCTGTGTTCCCTCGACTTGT	55.00	59.61		
*mTOR*	F: ATGAGCCAAGAGGAATCCAC	50.00	57.27	173	XM 006127044.3
	R: GACGCCATCTCCATGACGAC	60.00	60.87		
*S6k1*	F: GGTGCTTCAGCCAGTGCATCAA	54.55	63.91	101	XM 006138647.1
	R: GATGCCTCTCCGCAAACTGTCA	54.55	63.13		

*β-Actin*: beta-actin; *TNF-α*: tumor necrosis factor-α; *IL-1β*: interleukin-1β; *IL-10*: interleukin-10, according to Qiu et al. [41]; *mTOR*, mammalian target of rapamycin, according to Zhang et al. [42]; *IGF1*, insulin-like growth factor 1; *S6k1*, ribosomal protein S6 kinase 1.

**Table 4:** Effects of DGL on growth performance and feed utilization of *P. sinensis.*

Group	IBW	FBW	SR	WGR	SGR	FCR	FI
C	109.29 ± 3.06	143.9 ± 1.69 ^a^	95.00 ± 2.89	31.84 ± 3.41 ^a^	0.49 ± 0.05 ^a^	2.53 ± 0.20 ^b^	2.01 ± 0.02
G200	112.82 ± 2.46	151.54 ± 3.9 ^ab^	100	34.36 ± 2.95 ^a^	0.53 ± 0.04 ^a^	2.28 ± 0.18 ^ab^	1.96 ± 0.04
G400	110.20 ± 4.01	161.27 ± 5.4 ^b^	100	46.48 ± 3.74 ^b^	0.68 ± 0.05 ^b^	1.86 ± 0.10 ^a^	2.00 ± 0.06

Values are represented as the mean ± SEM (n = 3). Mean values in the same row with different superscript letters are significantly different (*p* < 0.05). IBW, initial mean body weight. FBW, final mean body weight. SR, survival rate. WGR, weight gain rate. SGR, specific growth rate. FCR, feed conversion ratio. FI, feed intake.

**Table 5:** Effects of DGL on morphometric parameters of *P. sinensis.*

Group	HSI	CF	TL/TW	TL/TH	TW/TH
C	3.12 ± 0.22	14.82 ± 0.51	1.17 ± 0.01	3.22 ± 0.1	2.76 ± 0.12
G200	2.91 ± 0.07	15.12 ± 0.21	1.15 ± 0.01	3.34 ± 0.09	2.90 ± 0.08
G400	3.27 ± 0.23	15.13 ± 1.08	1.15 ± 0.03	3.48 ± 0.06	3.02 ± 0.02

Values are represented as the mean ± SEM (n = 3). HSI, hepatosomatic index. CF, condition factor. TL, trunk length. TW, trunk width. TH, trunk height.

**Table 6:** Effects of DGL on body and hepatic chromaticity of *P. sinensis.*

Body Part	Color Intensity Parameter	C	G200	G400
Carapace	Lightness (L*)	31.22 ± 0.62	31.38 ± 1.74	30.99 ± 1.3
	Redness (a*)	1.99 ± 0.19	1.86 ± 0.3	1.89 ± 0.39
	Yellowness (b*)	6.18 ± 0.59	5.5 ± 0.99	5.94 ± 0.92
Plastron	Lightness (L)	69.81 ± 1.54	71.28 ± 0.79	69.4 ± 0.4
	Redness (a*)	4.17 ± 0.23	4.54 ± 1.22	5.28 ± 0.28
	Yellowness (b*)	13.84 ± 1.59	13.36 ± 1.51	12.75 ± 0.56
Liver	Lightness (L)	31.97 ± 0.27	30.48 ± 1.55	29.88 ± 0.67
	Redness (a*)	8.6 ± 0.32	9.88 ± 0.58	8.28 ± 0.47
	Yellowness (b*)	8.88 ± 0.26 ^b^	7.77 ± 0.64 ^ab^	6.12 ± 0.65 ^a^

Values are represented as the mean ± SEM (n = 3). Mean values in the same line with different superscript letters are significantly different (*p* < 0.05).

**Table 7:** Effects of DGL on serum biochemistry indexes of *P. sinensis.*

Parameter	C	G200	G400
Glu	3.88 ± 0.58	3.12 ± 0.59	2.33 ± 0.47
ALT	1.27 ± 0.09 ^b^	0.63 ± 0.03 ^a^	0.87 ± 0.27 ^ab^
AST	69.87 ± 2.10 ^b^	59.63 ± 1.59 ^ab^	48.17 ± 6.09 ^a^
ALP	481.93 ± 10.04 ^c^	350.17 ± 14.98 ^b^	251.23 ± 23.56 ^a^
LDLC	3.45 ± 0.69 ^b^	2.28 ± 0.27 ^ab^	1.67 ± 0.44 ^a^
HDLC	0.85 ± 0.01 ^b^	0.64 ± 0.02 ^a^	0.68 ± 0.04 ^a^
TC	3.75 ± 0.51 ^b^	2.13 ± 0.13 ^a^	2.09 ± 0.04 ^a^
ALB	14.82 ± 2.56	12.51 ± 0.78	11.29 ± 1.1
TP	27.62 ± 2.15 ^b^	26.17 ± 0.95 ^b^	21.15 ± 0.54 ^a^
TG	1.25 ± 0.07 ^b^	1.08 ± 0.01 ^a^	1.03 ± 0.02 ^a^
LPS	0.081 ± 0.005 ^b^	0.061 ± 0.004 ^b^	0.039 ± 0.008 ^a^

Values are represented as the mean ± SEM (n = 3). Mean values in the same line with different superscript letters are significantly different (*p* < 0.05). Glu, glucose. ALT, alanine aminotransferase. AST, aspartate aminotransferase. ALP, alkaline phosphatase. LDLC, low-density lipoprotein cholesterol. HDLC, high-density lipoprotein cholesterol. TC, total cholesterol. ALB, albumin. TP, total protein. TG, triacylglycerols. LPS, lipopolysaccharide.

## Data Availability

All data generated and analyzed during this study are included in this published article.
